# Supplementary material for: The Mechanisms of Thiosulfate Toxicity against Saccharomyces cerevisiae
Source: Antioxidants (Basel). 2021 Apr 22;10(5):646. doi: 10.3390/antiox10050646 (PMC8146336; doi:10.3390/antiox10050646)
Supplement: Supplementary file 1 [file antioxidants-10-00646-s001.zip › antioxidants-1154016-supplementary.pdf]

Supplementary information

The mechanisms of thiosulfate toxicity against *Saccharomyces cerevisiae*

Figure S1: The lethal effect of sulfur species on *S. cerevisiae* at different pH.

Figure S2: The release of H_2S from thiosulfate by yeast cells.

Figure S3: Thiosulfate tolerance of different *S. cerevisiae* strains.

Figure S4: The reaction of thiosulfate with Cu^{2+}.

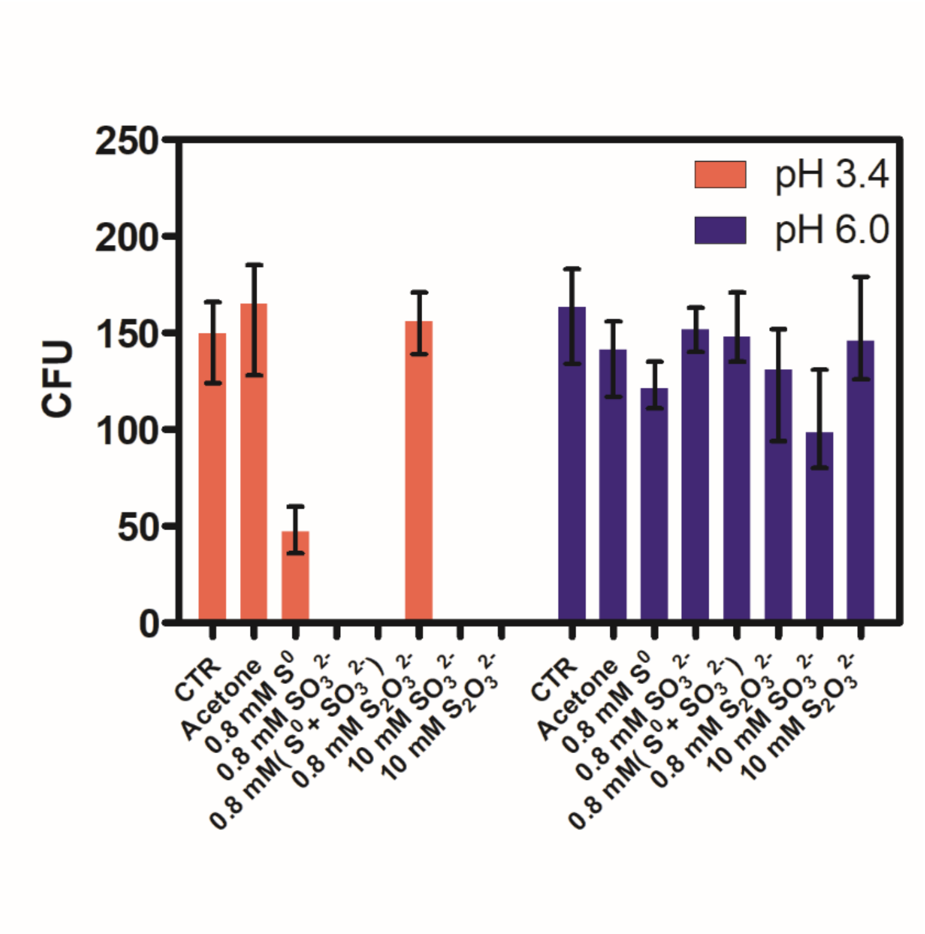

Figure S1: The lethal effect of sulfur species on *S. cerevisiae* at different pH. Equal number of yeast cells (OD_{600nm} = 1) were incubated in 1 mL citric acid-sodium phosphate dibasic buffer (pH 3.4 or 6) with S^0, sulfite, thiosulfate, or the combination of S^0 and sulfite at 30°C for 1 h. The suspensions were then diluted with sterile water by 10^4 times, 100 μL of dilutions was spread on the YPD plate and cultured at 30°C for two days. Then the CFUs (colony formation units) was calculated. CTR (control): yeast cells suspend in the buffer without sulfur addition.

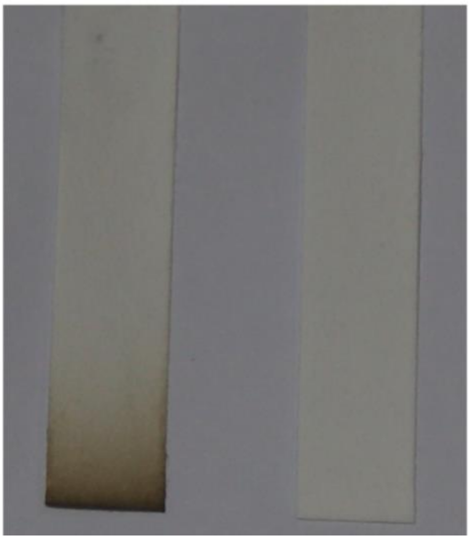

Figure S2: The release of H_2S from thiosulfate by yeast cells. The wild type and mutant (*RDL1* deletion strain) were subjected to sulfur starvation for two days. Thiosulfate was added to 1 mM to 3-ml cell cultures (OD_{600} of 1) and incubated for 2 h at room temperature. Detection of H_2S production in the gas phase was done with lead-acetate paper strips. Left: wild type; Right: *Δrdl1* strain.

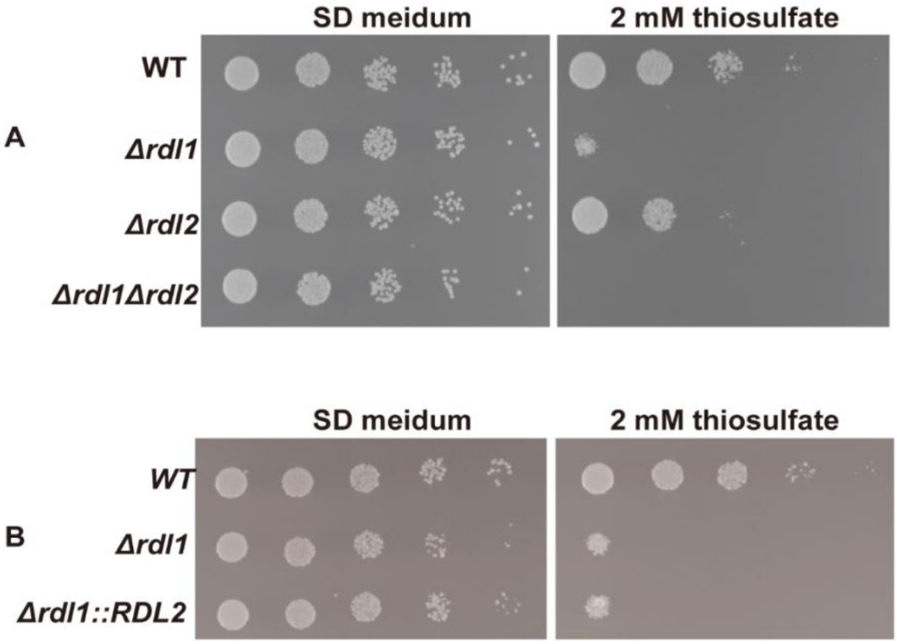

Figure S3: Thiosulfate tolerance of different *S. cerevisiae* strains. The wild type and the mutant strains incubated in SD plate or SD plate with 2 mM thiosulfate.

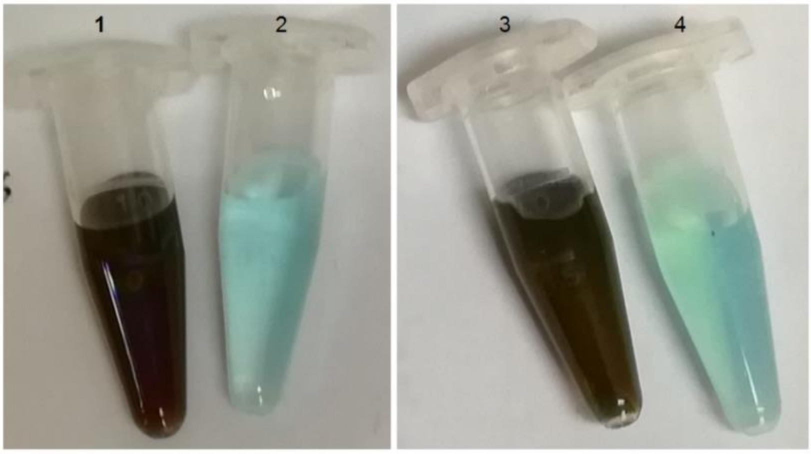

Figure S4: The reaction of thiosulfate with Cu^{2+}. 1&3) the mixture of 10 mM thiosulfate with 10 mM copper chloride at room temperature; 2&4) 10 mM copper chloride.

1&2) 50 mM Tris-HCl, pH 7.6; 3&4) 50 mM HEPES, pH 7.0.
